# Comprehending Nutrition and Lifestyle Behaviors of People with Metabolic Syndrome: A Focus Group Study

**DOI:** 10.3390/healthcare10091653

**Published:** 2022-08-30

**Authors:** Muhammad Daniel Azlan Mahadzir, Kia Fatt Quek, Amutha Ramadas

**Affiliations:** Jeffrey Cheah School of Medicine and Health Sciences, Monash University Malaysia, Jalan Lagoon Selatan, Bandar Sunway 47500, Malaysia

**Keywords:** metabolic syndrome, nutrition, lifestyle, qualitative study, focus group discussion

## Abstract

Demographically and socio-culturally appropriate care is critical for empowering people with metabolic syndrome (MetS) to self-manage their condition. This focus group study aimed to explore the understanding of nutrition and lifestyle behaviors (NLBs) of Malaysians with MetS. Adults with MetS (N = 21) participated in four focus groups at a university’s research clinic in Malaysia. A thematic framework analysis approach was applied to the focus group data using an initial coding framework developed from the Health Belief Model. Six main themes were identified on perceived motivations, barriers, and threats toward healthy NLBs. Motivations to adopt healthy NLBs were body image, personal experience of adverse complications, and family and social support. The perception that healthcare is a business model, the idea that changes in NLBs are difficult and expensive, and cultural influence on food intake were identified as barriers to healthy NLBs. Inadequate knowledge of MetS was identified as a subtheme in this study. Health education and health promotion activities that aim to modify the NLBs of people with MetS should consider the community’s perception of motivation and barriers to change. Addressing these aspects in the development of programs can potentially increase program adoption and adherence, ensuring the success of community-based lifestyle interventions.

## 1. Introduction

Nutrition and lifestyle behaviors (NLBs) have been associated with the risk of metabolic syndrome (MetS) [1]. MetS is a cluster of cardiovascular risk factors that places individuals at increased risk for type 2 diabetes and cardiovascular diseases [2,3]. The distribution of MetS is concentrated in high-economy countries [2] and developing countries, such as Malaysia [4]. In their nationwide study, Rampal et al. [5] found that the prevalence of MetS in Malaysia ranges from 25% to 41%. The increase in MetS prevalence among Malaysian adults over the last decade [4,5,6,7] reinforces the need for an effective health promotion program with demographically and culturally appropriate care information that addresses critical factors such as physiological function and social psychology. Such programs can empower these patients to enhance their self-care-related motivation and confidence.

Theoretical frameworks such as the Health Belief Model (HBM) have been widely used to explain an individual’s behaviors and behavioral changes, which are beneficial aspects of program development [8,9,10]. Four constructs of the HBM represent the perceived threat and net benefits (perceived susceptibility, perceived severity, perceived benefits, and perceived barriers), which account for an individual’s “readiness to act” [11]. The HBM has been further expanded to include two more constructs—cues to action and self-efficacy—to address challenges of changing unhealthy habitual behaviors, such as sedentariness, smoking, or overeating [8]. Given the usability of the constructs of the HBM to explain risky behaviors that are related to chronic diseases [9], it has been widely used to explain the findings in cross-sectional studies of MetS [12,13]. A recent study by Park and Kim [14] used the HBM as the guiding theory in their qualitative analysis. In addition, Hirakawa et al. [15] reported that the HBM can be used to explore the factors that cause individuals to neglect specific health recommendations.

A greater understanding of individuals’ motivation for and barriers to NLBs is critical for ensuring the success of lifestyle interventions [1]. As the current understanding of behavioral determinants that shape the NLBs of Malaysians with MetS is limited, we aimed to explore the NLBs of people with MetS by using the HBM as the theoretical framework. Through this study, we conducted and qualitatively analyzed a series of focus group discussions (FGDs) involving Malaysian adults with MetS to explore their understanding of MetS and their perceived motivation for and barriers to healthy NLBs.

## 2. Materials and Methods

### 2.1. Study Design and Setting

A focus group study was performed with individuals with MetS. The qualitative nature of the study was essential in order to ensure a more in-depth exploration of the motivation for and barriers to behavioral change. The study was conducted according to the guidelines of the Declaration of Helsinki and approved by the Monash University Human Research Ethics Council (MUHREC) before commencement (Project ID: CF16/56—2016000022).

### 2.2. Respondents

Adults (>18 years) attending a university research clinic in Malaysia were invited to participate in the study. Participants were recruited using purposive sampling; they were sought out based on their MetS status, which was the focus of the study. All consenting patients who fulfilled the Harmonized Criteria [16] for MetS were invited to join the FGDs, which were led by a moderator. Sociodemographic details and MetS components were recorded for all participants. The total number of FGDs was determined based on data saturation. This is a concept in which we could no longer synthesize new information, as the discussion’s topic or theme repeated in each FGD. Hence, we halted the recruitment once the FGDs no longer provided any new data or information for this study. 

### 2.3. Focus Group Discussions

Four FGD sessions involving 21 participants took place in a private consultation room and were led by a moderator (M.D.A.M.), who was accompanied by a trained assistant. The responsive interviewing model [17] and HBM framework [18] were used to develop the interview protocol. As the researchers were interested in understanding the study patients’ interactions with their peers within the focus group, the group dynamics and interactions were enhanced by reassuring the study patients that (i) their confidentiality was guaranteed, (ii) there were no ‘right’ or ‘wrong’ answers to the questions, and (iii) constructive criticism was a valued part of the process. The FGD guide is provided in Supplementary Table S1. 

### 2.4. Analyses

All FGD session recordings were transcribed verbatim before undergoing thematic analysis. Some respondents spoke in Malay or a combination of English and Malay. Hence, the conversations were directly translated into English for the purposes of analysis. However, we ensured that the original meanings of these conversations remained as accurate as possible. The steps of the thematic framework analysis [17,18,19] were implemented using a priori issues derived from the constructs of the HBM. Two HBM components—motivation and perceived barriers toward healthy NLBs—were used as the primary domains in the thematic framework to facilitate textual, structural, and composite descriptions of NLBs among Malaysian adults with MetS. Themes were developed by comparing codes within each category, which was done to ensure that the interpretations remained grounded in the themes and codes. We explored the relationship by evaluating the frequency of overlapping themes in the excerpts. This relationship explains the connections between codes and helps us to explain our main findings and highlight any overarching themes. 

## 3. Results

### 3.1. Characteristics of Study Respondents

The average age of the study patients (n = 21) was 51.0 (SD = 10.3) years old, and they ranged in age from 26 to 64 (Table 1). The majority were women (71.4%) and married (71.4%), and all had equal to or more than a high school education (76.2%) and were employed full-time (71.4%). All participants were hypertensive and had abdominal obesity as defined by the Harmonized Criteria [16].

### 3.2. Themes

**Figure 1:**
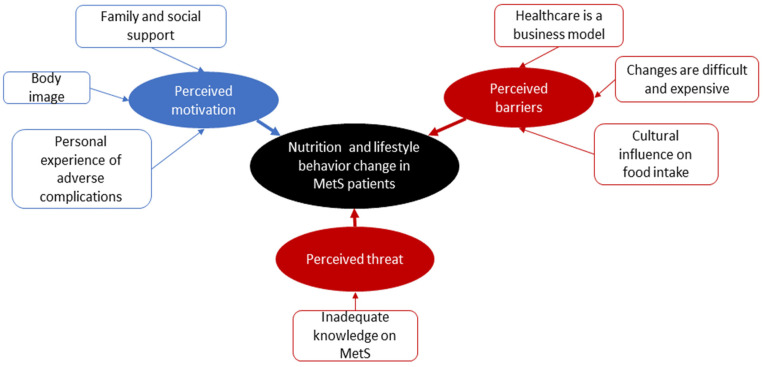
Identified themes in the thematic analysis of the focus group discussions.

Seven themes relating to healthy NLB changes emerged from the qualitative analysis. Figure 1 summarizes the themes identified in the focus group study. 

#### 3.2.1. Perceived Motivation to Adopt Healthy Nutrition and Lifestyle Behaviors

**Theme 1: Body Image**

We found body image perception to influence the MetS patients’ lifestyle changes (Table 2). While some patients reported a tendency towards anti-aging products and food supplements to counter excess weight gain, six extended their health monitoring by improving dietary habits and increasing physical activity. Female MetS patients were more concerned about body image—specifically, skin and physical beauty. Observable changes in their skin prompted the female patients to eat healthier and be physically active. Weight gain was also seen as a health threat among male patients.

**Theme 2: Personal Experience of Adverse Complications**

The study patients shared their fears that being in poor health may not allow them to continue their everyday roles and how disability may negatively affect their lives. The experience of having or caring for close family and relatives who had post-stroke paralysis and how this has inversely affected their quality of life reportedly motivated some of them to change their NLBs. The patients expressed that they did not want their health to be compromised and resulted in them being unable to provide care for themselves or their families.

**Theme 3: Family and Social Support**

The FGD participants were asked about the environment and surroundings that may affect their life choices and habits. Eight participants voiced the need for support when making lifestyle changes. The support sought was received in many forms in the past. For example, some participants shared how specific individuals and pets had supported them positively and boosted positive behavioral changes. We must understand that NLB changes can be incredibly challenging without support from family or the closest individuals. This issue was observed in one of the patients who shared her experience in which her spouse prevented her from going for daily walking, as he was intimidated by her healthy lifestyle. 

#### 3.2.2. Perceived Barriers toward Healthy Nutrition and Lifestyle Behaviors

**Theme 4: Healthcare as a Business Model**

We found a complex relationship between the MetS patients and their healthcare providers (Table 3). Most participants had been regular visitors to the university research clinic for more than two years. They reemphasized the importance of trust between them and healthcare providers and described the experience of doctors prescribing medications without many consultations. Five participants disclosed that they were more interested in non-pharmacological options, such as dieting and exercise, in order to reduce the risk of their diseases. Unfortunately, previous healthcare providers placed little emphasis on dieting and exercise behaviors, and they perceived that doctors were rushed during visits.

Moreover, several of the interviewed patients voiced concerns that some illnesses were being manipulated to financially benefit the pharmaceutical industry. These statements portrayed a lack of understanding among the study patients on MetS and its clinical management. All participants viewed good communication and personal contact as building blocks for establishing trust in the patient–provider relationship.

**Theme 5: Changes in Nutrition and Lifestyle Behaviors Are Difficult and Expensive**

Although we found the participants to be generally well informed about good dietary habits, such as the food pyramid, food timing, and calorie counts, they tended to struggle to apply them in their daily lives. Participants shared their experience of relapsing into their old habits after making several NLB changes, as they found the changes too tricky and drastic. The FGDs yielded a few factors that may have inhibited the patients’ adoption of NLBs—(i) lack of support for practising of a healthy diet and exercise, (ii) tedious process of calorie counting, (iii) affordability, and (iv) availability of healthy foods. The patients viewed changing eating and physical activity for the long term as a “battle” and something that they had to control. The discouragement of relapse came through very clearly in all FGD sessions.

**Theme 6: Cultural Influence on Food Intake**

Generally, the patients assumed that consuming a plate filled with vegetables and organic foods and drizzled with olive oils reflects a healthy diet. Most participants recognized the ‘Mediterranean Diet’ as the perfect healthy diet. Thus, most of them regarded local foods as “cheat meals.” Several patients shunned healthy eating due to a preconceived idea that rice must be avoided, which is almost impossible in the Asian community.

#### 3.2.3. Perceived Threat towards the Adoption of Healthy Nutrition and Lifestyle Behaviors

**Theme 7: Inadequate Knowledge of the Metabolic Syndrome**

Patients could identify the components of MetS individually and relate them to their poor lifestyle and dietary habits (Table 4). However, the participants were unfamiliar with MetS as a clustered disorder, as their respective health providers did not inform them of such a condition. However, the participants were aware of the necessity of NLB changes in order to improve general health, such as by using the Malaysian healthy serving plate concept: Quarter, Quarter, and Half [20]. However, they expressed a need for skill-building to take corrective actions to ensure the success of behavioral changes that would reduce their risk of MetS. 

## 4. Discussion

We identified body image as one of the strongest motivations for adopting healthy NLBs. Gradual weight gain experienced in middle age increases the risk of fracture and disability and reduces the quality of life [21,22,23]. Specifically, abdominal obesity raised concern among the study patients, resulting in poor body image. Our finding suggests that interventions that focus on physical changes such as weight loss may be perceived to be more favorable than using blood parameters as targets. Hence, targeted interventions may exploit this concern by addressing the benefit of weight loss in terms of physical appearance and healthy aging [22].

We also found individuals with MetS who had experienced adverse health complications to be highly motivated to adopt a healthier lifestyle. Past qualitative studies conducted among stroke [24,25] and dialysis patients [26] also found a relationship between experiences with health complications and subsequent life choices. In addition, we found that respondents with family members affected by health complications tended to adopt a healthier lifestyle. Similar findings were reported previously, as the individuals tended to change their behaviors after observing the poor quality of life and effects on their family members [27,28]. As a result, lifestyle intervention may be a significant step in managing MetS [2,29,30,31]. 

Having a sound support system was another perceived motivation for NLB changes. We discovered that individuals residing in a neighborhood with an active community body tended to engage in weekly physical activity. In addition, living in surroundings with public parks, a safe pedestrian walkway, and a gymnasium was likely to increase the physical activity levels of MetS patients. Adoption of physical activity as a part of NLBs is more likely to be influenced by family members [25,32]. In addition to family members, supportive peers were also reported to be among the reasons that our respondents adopted better NLBs. Peer support studies demonstrated that peers have a substantial influence on the behavioral change of others, as they share similar health conditions and usually have similar living surroundings [30,31,32,33,34,35]. 

The perception that healthcare—mainly preventative and primary care—is a business model was one of the perceived barriers identified in this study. The patients in the study argued that preventative medicine and primary care intentionally create the need to seek early but unnecessary medical attention that benefits certain industries, such as pharmaceuticals and nutraceuticals. This finding raised a red flag concerning a general misunderstanding that could hinder the reach of primary healthcare in the community. Similar findings were reported in India [36] and Singapore [37], where healthy older adults reported avoiding regular check-ups due to the fear of spending more on unnecessary healthcare. Thus, future interventions must overcome this barrier by providing individuals with correct and crucial information about chronic diseases. The findings from our FGDs also demand interventions that require healthcare professionals to be more sensitive to the needs of their patients. This includes having sufficient knowledge of NLBs that would suit a specific target population—for example, the prescription of low-impact physical activity to overcome the sedentary lifestyles of older adults. 

The FGD participants also perceived that adoption of NLB change is a difficult task and can be expensive. Their concerns revolved around healthy meal preparation, healthy food choices while shopping or eating out, and increasing physical activity on a busy workday. Furthermore, we found that healthy foods were understood as green choices, organic products, and non-GMO products instead of food groups and varieties. Hence, the participants concluded that healthy foods were expensive, as shown in previous studies [38,39]. Solbrig et al. [40] reported the limitation of understanding and translating available health information into accessible and practical steps in daily life to be the significant barrier to adopting these long-term changes. 

Malaysians exhibit different preferences in terms of food choices and lifestyle behaviors, primarily due to the multicultural background of the nation’s people. Local population studies reported that cultural background influences individuals’ food choices [5,41]. For example, the Malay community generally consumes large amounts of fats and carbohydrates [5,41], while the Chinese community is more receptive to a healthy lifestyle than other ethnicities are [5,6]. Nationwide epidemiologic studies have shown the prevalence of MetS to vary according to ethnicity [5,6,41], and as we have identified that cultural influences could be a barrier to adopting specific NLB changes that we would recommend, a clear understanding of each community’s cultural beliefs is crucial in the intervention development. 

Finally, most participants were unaware of MetS as a cluster of risk factors, but could identify the components individually. This corresponds with the HBM, as it is suggested that the three main initiators of healthy lifestyle choices are motivation to change, perceived barriers to changes, and perceived threat of diseases [42]. The study respondents were uninformed about MetS as a threat, as the components of MetS were viewed individually instead of being concerned with their ‘clustering’. However, although the management of MetS targets each component individually, information on the threats posed by the clustering of risk factors may improve the perception of change among individuals, as reported in a previous study [43].

The findings from this study were used to inform the delivery approach and information provided to individuals in a community-based peer support program, PERSUADE [30,31]. Since this focus group study identified social support as one of the essential elements in influencing healthy NLBs among adults with MetS, it has improved the relevance of the peer support construct in interventions for adults with MetS. In addition to a scoping review finding that explored the usability of a group-based NLB intervention [44], social support delivered by trusted individuals, such as peers, will improve the rate of adoption and retention of healthy NLB changes. 

This study has a notable limitation in term of participants sociodemographic distribution, as most of the respondents were female and had a high level of education. Additionally, the study only provided a perspective of the urban community where the recruitment was done. With this in mind, we acknowledge that there might be different motivations and barriers for different socioeconomic communities. A more robust qualitative study with more individuals from different socioeconomic backgrounds might reflect better perspectives on NLB motivations and barriers. From another perspective, a qualitative study with an a priori construct based on an existing behavioral model may be limited, as the discussion and subsequent analysis were constrained within this proposed construct [45]. Arguably, the HBM is a dated behavioral model and although it is still relevant, a more recent construct can also be tested [46,47]. Future studies may explore the perspectives of healthcare providers concerning NLB-related health education for patients with chronic diseases, such as MetS. 

## 5. Conclusions

Collectively targeting MetS warrants a holistic point of view. Interventions must be specifically designed with respect to the need for change by delivering a basic understanding of diseases, their severity, and individuals’ susceptibility to acquiring them. The thematic analysis of FGDs in this study informed about the perception of change in Malaysian adults towards a healthy lifestyle. Individual perceptions of disease threat, body image, adverse health effects, the healthcare system’s reliability, and the benefits of changes significantly modulate the need to change. In addition, a supportive environment will increase the adoption and sustainability of a healthy lifestyle. In conclusion, the results support the practicability and value of this study in informing the development of better lifestyle interventions targeting Malaysian adults with MetS.

## Figures and Tables

**Table 1:** Characteristics of the study respondents (N = 21).

Characteristics		Mean (SD)	n (%)
Age		51.0 (10.3)	
Sex	Male		6 (28.6)
	Female		15 (71.4)
Marital status	Single		1 (3.4)
	Married		15 (71.4)
	Widowed/separated		5 (23.8)
Education level	Secondary		5 (23.8)
	Tertiary		16 (76.2)
Occupation	Employed/self-employed		15 (71.4)
	Unemployed		1 (4.7)
	Retired		5 (23.8)
Personal income (MYR)	<2000		3 (14.3)
	2000–3999		5 (23.8)
	4000–5999		5 (23.8)
	>6000		8 (38.1)
Metabolic risk factors	Abdominal obesity		21 (100.0)
	Hypertension		21 (100.0)
	Diabetes		19 (90.5)
	Dyslipidemia		7 (33.3)
	Hypertriglyceridemia		9 (42.9)

Note: 1 USD = MYR 4.40 (June 2022).

**Table 2:** Themes derived from responses given by study respondents (N = 21) in the domain of perceived motivation.

Themes	Example of Responses
Body image	*“You can eat all the pills, but you will see the difference only once you sweat and eat better”.* *“The tummy has become large until I feel tired carrying it”.* *“I only started exercising when my dermatologist said it helps get rid of my wrinkles”.*
Personal experience of Adverse complication	*“I’ve made up in my mind, but I’m not ready to go yet as I am not sure how to start.”*
	*“A couple of years ago, my father had a stroke at 59 years old—could not move, could not eat, could not do anything, and depend on us (the children)...it really started all of us thinking and decided to change. Whatever goes inside my husband’s and my children’s mouths are taken care of. It will be too late when we get a stroke. Why want to trouble everyone around us because of our bad habits…”*
Family and social support	*“I think that means a lot when you got somebody else in the house that’s take care of food and drinks and conscious about food every day”.* *“I always join my neighbor to walk around the neighborhood. Usually, we will do it in a group. I feel very moved to go for a walk. My wife and kids are together too. On weekends, we usually have a barbeque get-together. That kind of support makes me feel better about myself.”* *“My husband is a jealous type. If he does not like it, you better not do it. Like walking and all, he can never see me doing it. Because when I do it, and he is lazy, he feels intimidated.”*

Note: Verbatim translation of respondents’ responses during the FGDs.

**Table 3:** Themes derived from responses given by the study respondents (N = 21) in the domain of perceived barriers.

Themes	Example of Responses
Healthcare as a business model	*“I always heard ‘you are what you eat’, but doctors are busy prescribing medications only.”* *“…sometimes when the doctors prescribed too many medications, they act as they work for the pharmacy.”* *“I think doctors give too much medication until five or six different medicines will cancel out something.”*
Difficult and expensive nutrition and lifestyle behavior changes	*“I bought all the weight loss vitamins (supplements) for nearly 10,000 (MYR), it feels slimmer for a while, but after a year, I feel my body expands.”* *“Once you have been off a diet and go back, it’s just harder to go back.”* *“Health needs discipline. That is why a lot of successful people have a healthy life. They have the discipline to be healthy.”*
Cultural influence on food intake	*“We as Malaysians can never stop eating rice. Make rice healthy and then only tell us to diet.”* *“We are told what to eat since we are kids. If you are Chinese, you will eat more soup and not eat at night. It’s different from Indian, Malay and any other race in Malaysia.”*

Note: Verbatim translation of respondents’ responses during the FGDs.

**Table 4:** Themes derived from responses given by the study respondents (N = 21) in the domain of perceived threats.

Themes	Example of Responses
Inadequate knowledge of metabolic syndrome	*“We only know about diabetes, high blood pressure, and high blood cholesterol”.* *“We were only told to eat properly, exercise, and eat our medication. Metabolic syndrome is new. If you said it’s the big tummy problem, that’s what I call fat.”*

Note: Verbatim translation of respondents’ responses during the FGDs.

## Data Availability

Raw data, including the recordings and verbatim translations of the focus group discussions, cannot be made available due to ethical restrictions. The data summarized in this study are available from the corresponding author upon request.

## Supplementary Materials

The following are available online at https://www.mdpi.com/article/10.3390/healthcare10091653/s1, Table S1: Focus group discussion guide.
