# Supplementary material for: Comprehending Nutrition and Lifestyle Behaviors of People with Metabolic Syndrome: A Focus Group Study
Source: Healthcare (Basel). 2022 Aug 30;10(9):1653. doi: 10.3390/healthcare10091653 (PMC9498472; doi:10.3390/healthcare10091653)
Supplement: Supplementary file 1 [file healthcare-10-01653-s001.zip › healthcare-1861740-supplementary.pdf]

Table S1. Focus Group Discussion Guide

No	Question	Probe
1	What do you understand about a healthy diet?	• Do you think you are consuming a healthy diet?<br>• Aware of the importance of a healthy diet?<br>• Do you think foods are making you sick?<br>• What is the hardest thing to achieve in a healthy diet?
2	How many times do you eat every day?	• What do you usually eat every day?<br>• If you can skip, which meal do you skip the most?<br>• How about breakfast, is it important?<br>• Would you skip lunch or breakfast?<br>• Dinner? When is your dinner?<br>• Do you usually eat *kuih* or snacks? How about while watching TV?
3	How to get enough nutrients for a healthy diet?	• Do you prioritize fruits and vegetables?<br>• Are you limiting carbohydrates like rice? (if yes, how?)<br>• What is your opinion of food supplements? Do you take them?
4	What kind of exercise do you like the most?	• Do you exercise every day? How long?<br>• Do you do it alone or with the family?<br>• Is there a park or ground near your house or do you prefer to go to the gym? Why?<br>• Football or futsal? Jogging or cycling?
5	Does any of your family members smoke cigarettes in the house?	• Do you smoke? Did your parents smoke? (if yes) Are you smoking because your parents did?<br>• What does your workplace look like? Do you smoke with a friend or colleague?<br>• When do you usually smoke the most? How about the afternoon or after a meal like lunch?
6	Have any of you drunk alcohol before?	• (if yes) are you a regular?<br>• Describe to me the best liquor in the market.<br>• Do you usually get drunk?<br>• Do you prefer wine or beer?
7	Are you happy with your sleep?	• Do you wake up fresh the next day?<br>• Any nightmare?<br>• Do you snore? (if yes), Since when do you snore? Disturbed?<br>• Do you usually feel sleepy after a meal?<br>• Do you take a nap or fall asleep after a meal?<br>• How long do you think the best sleep should be?
8	How do you describe your neighbor?	• Are you close with them?<br>• How many family members do they have? What is their occupation?<br>• Is there any neighborhood community in your area?<br>• Do you usually do activities in your neighborhood?<br>• What main thing do you want to improve around you?
9	Do you have relatives close by?	• Frequent visits to their house?<br>• Do they always come to help you?
10	With the size of the family, is it a lovely house to live in now?	• Do you like your current address now?<br>• Do you have any intention of moving?<br>• Are there closeby hospitals, clinics, and malls to you?<br>• Do you always go for a medical check-up? (if yes), Why?<br>• Do you wish your family stays with you?
